# Associations between Knowledge of Health Risks and Sugar-Sweetened Beverage Intake among US Adolescents

**DOI:** 10.3390/nu15102408

**Published:** 2023-05-22

**Authors:** Sohyun Park, Seung Hee Lee, Caitlin Merlo, Heidi M. Blanck

**Affiliations:** 1 Division of Nutrition, Physical Activity, and Obesity, National Center for Chronic Disease Prevention and Health Promotion, Centers for Disease Control and Prevention, Atlanta, GA 30341, USA; xde5@cdc.gov (S.H.L.); hcb3@cdc.gov (H.M.B.); 2 Division of Overdose Prevention, National Center for Injury Prevention and Control, Centers for Disease Control and Prevention, Atlanta, GA 30341, USA; 3 Division of Population Health, National Center for Chronic Disease Prevention and Health Promotion, Centers for Disease Control and Prevention, Atlanta, GA 30341, USA; ihb7@cdc.gov

**Keywords:** sugar-sweetened beverages, knowledge, adolescents, health conditions, behavior

## Abstract

Background: Sugar-sweetened beverage (SSB) intake is associated with adverse health outcomes. Objective: We examined associations between the knowledge of health risks related to SSB and SSB intake among adolescents. Design: A cross-sectional study using 2021 YouthStyles survey data. Participants/settings: 831 US adolescents (12–17 years old). Main outcome measures: The outcome variable was SSB intake (none, 1–6 times/week, and ≥1 time/day). Exposure variables were knowledge of seven SSB-related health risks. Statistical analyses performed: Seven multinomial regressions were used to estimate adjusted odds ratios (AOR) for drinking SSB, according to knowledge of SSB-related health risks and after controlling for sociodemographics. Results: Overall, 29% of adolescents consumed SSB ≥1 time/day. Although most adolescents identified cavities (75.4%), weight gain (74.6%), and diabetes (69.7%) as being related to drinking SSB, fewer adolescents identified related conditions such as high blood pressure (31.7%), high cholesterol (25.8%), heart disease (24.6%), and some cancers (18.0%). Compared to non-SSB consumers, drinking SSB ≥1 time/day was significantly higher among adolescents who lacked knowledge of associations between SSB intake and weight gain (AOR = 2.0), heart disease (AOR = 1.9), or some cancers (AOR = 2.3) after controlling for covariates. Conclusions: Among US adolescents, knowledge of SSB-related health risks varied by condition, ranging from 18% (some cancers) to 75% (cavities and weight gain). There were increased odds of drinking SSB among those unaware that weight gain, heart disease, and some cancers are associated with SSB intake. Intervention could evaluate whether increasing certain types of knowledge may influence youth SSB intake.

## 1. Introduction

Sugar-sweetened beverages (SSB) are the leading sources of added sugars in the diet of American adolescents [1,2]. Frequent consumption of SSB is associated with numerous adverse health consequences [3] such as obesity [4,5], type 2 diabetes [6,7], cardiovascular disease [7,8,9], cavities [10,11], high blood pressure [12,13], dyslipidemia [14,15], and cancer [16,17,18]. SSB include, but are not limited to, non-diet sodas, fruit-flavored drinks (that are not 100% juice), sweetened coffee or tea drinks, sports drinks, energy drinks, and other beverages that are sweetened with different forms of added sugars [19]. Adolescents are one of the high consumers of SSB in the United States [20], and high consumption of SSB is a public health concern due to the aforementioned adverse health consequences.

In addition to sociodemographic factors related to SSB intake among US adolescents [21,22,23,24,25], there are various behavioral and other factors associated with SSB intake such as attitudes [26], use of social networking sites [27], parent SSB intake [26,28], and availability of SSB at home [29,30]. For instance, the odds of adolescents consuming SSB at least once per day were 3.3 times greater among adolescents who had parents consuming SSB at least twice per day compared to adolescents whose parents did not consume SSB during the past month [28]. Another study reported that the odds of high SSB intake (≥2 times/day) were 5.6 times greater among adolescents who often or always had SSB available at home compared to those who never had SSB at home [30]. However, study findings on nutritional knowledge and SSB intake among adolescents are inconsistent. An Australian study found that knowledge of health risks (diabetes, weight gain, heart disease, tooth decay, and cancer) was associated with soft drink intake among students aged 12–17 years [31], while other studies reported that knowledge of SSB-related health risks in youth or parents/caregivers (weight gain, diabetes, and cavities) were not related to daily SSB intake among US youth [26,28]. These inconsistent findings from previous studies on youth call for additional research. Thus, we examined associations between knowledge of health risks related to SSB and SSB intake among US adolescents.

## 2. Methods

### 2.1. Study Sample and Survey Administration

We conducted a cross-sectional study using data from the summer wave of Porter Novelli Public Services’ Styles surveys, which are online panel surveys via Ipsos’ KnowledgePanel, representative of the noninstitutionalized US population [32]. Panel members are recruited by mail using a probability-based sampling method by address. If needed, a laptop or tablet and/or Internet access was provided. The panel members are continuously recruited, and their number kept at approximately 60,000 panelists. The survey asks about various topics including health-related knowledge, behaviors, and attitudes.

**Figure 1:**
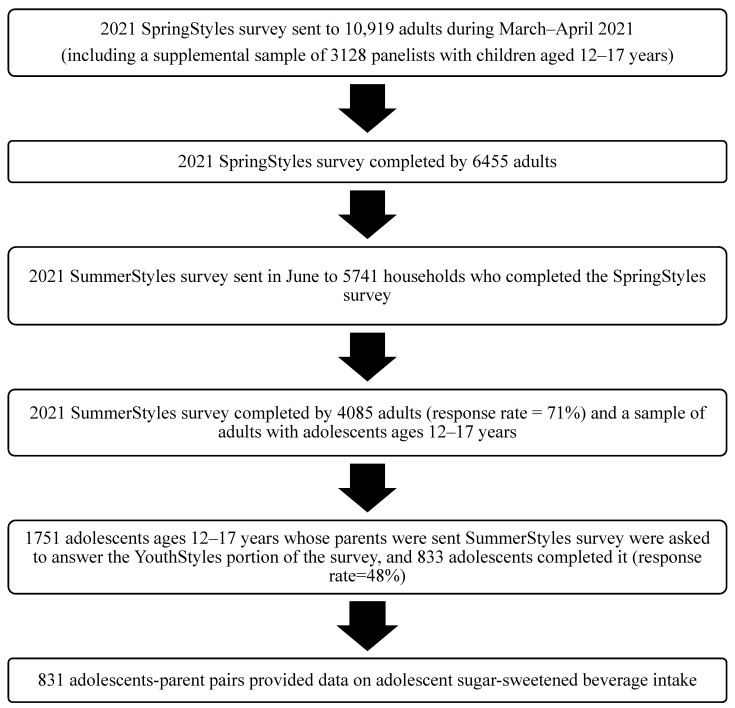
Analytic sample flow chart for SummerStyles and YouthStyles surveys among US adolescents and their parents/caregivers, 2021.

As illustrated in Figure 1, the SummerStyles survey was sent to participants in June 2021 who completed the SpringStyles survey, which is the initial wave. From March 2021 to April 2021, the SpringStyles survey was sent to 10,919 panelists aged ≥18 years, including a sample of 3128 panelists with children aged 12–17 years (to ensure pair cases for the SummerStyles survey). Of those, 6455 adults completed the SpringStyles survey (response rate of 59%). Ipsos sends the minimum number of invites needed to achieve desired sample size for each survey. The SummerStyles survey was sent in June 2021 to 5741 adults who completed the SpringStyles survey, and 4085 adults completed it (response rate of 71%). In addition, 1751 adolescents ages 12–17 years (whose parents received the SummerStyles survey) were asked to answer the YouthStyles portion of the survey, and 833 adolescents completed it (response rate of 48%). The YouthStyles data weights, which are used in this analysis, were based on the SpringStyles adult weights and then adjusted for the number of adolescents in the household, youth age, sex, and race or ethnicity, household income, census region, and metropolitan status. Of those 833 adolescents who completed the YouthStyles survey, we excluded from analysis 2 adolescents with missing data on an outcome variable (SSB intake) from this analysis, leaving a final analytic sample of 831 adolescents and their parents or caregivers.

Parents participated in their survey portion immediately before their child’s survey participation and provided electronic consent for their child to participate. Youth-adult dyad households who completed the survey received 10,000 cash-equivalent reward points (worth approximately $10) to be split between the parent and youth respondents. Respondents were not required to answer individual questions and could exit the survey at any time. Because the data provided to the Centers for Disease Control and Prevention (CDC) did not include personal identifiers, this study was exempt from the CDC Institutional Review Board.

### 2.2. Outcome Variables

The outcome variable was the self-reported frequency of adolescent SSB intake, which was measured using the following question: “During the past 7 days, how many times did you drink sodas, fruit drinks, sports or energy drinks, and other sugar-sweetened drinks? Do not include 100% fruit juice or diet drinks.” Response options were none, 1–6 times/week, 1 time/day, 2 times/day, 3 times/day, ≥4 times/day. To assess daily SSB intake, we created three mutually exclusive categories (none, 1–6 times/week, and ≥1 time/day).

### 2.3. Exposure Variables and Covariates

The exposure variables were adolescent knowledge of seven SSB-related health risks—cavities, weight gain, diabetes, high blood pressure, high cholesterol, heart disease, and some cancers. The following questions were used: “Which of the following conditions do you think are related to drinking sugary drinks, such as regular sodas, fruit drinks (e.g., Kool-Aid, lemonade), sports or energy drinks (e.g., Gatorade, Red Bull), and sweetened teas?” Respondents were given the following health risks and asked to choose all that apply: cavities, weight gain, diabetes, high blood pressure, high cholesterol, heart disease, some cancers, or none of these.

Covariates included sociodemographic factors for both adolescents and their parents/caregivers. For adolescents, we included age (12–14, 15–17 years), sex (male, female), and race or ethnicity (non-Hispanic [NH] Black, Hispanic, NH Other/Multiracial, or NH White) as covariates. For the responding parents/caregivers, we included parent age (18–34, 35–44, or ≥45 years, consistent with previous studies [26,28]), sex (male, female), race or ethnicity (NH Black, Hispanic, NH Other/Multiracial, or NH White), education (≤high school/GED, some college, or ≥college graduate), marital status (married/domestic partnership, not married), annual household income (<$35,000, $35,000–$74,999, $75,000–$99,999, or ≥$100,000), census regions of residence (Northeast, Midwest, South, or West), and parent SSB intake during the past month (0, >0 to <1, 1 to <2, ≥2 times/day).

### 2.4. Statistical Analysis

For bivariate analysis, we used chi-square tests, and a *p*-value ≤ 0.05 was considered statistically significant. For multivariate analysis, we used multinomial logistic regression models to estimate adjusted odds ratios for adolescent SSB intake ≥1 time/day and 1–6 times/week, using none as a reference. Regression models were fit for each of the seven exposure variables of adolescent knowledge (yes or no) of health conditions related to SSB intake (cavities, weight gain, diabetes, high blood pressure, high cholesterol, heart disease, and some cancer) due to collinearity of exposure variables. Each regression model controlled for adolescent age, sex, and race or ethnicity as well as parent age, sex, race or ethnicity, education, marital status, annual household income, census regions of residence, and parent SSB intake. Of those 831 adolescents with outcome data, the sample size was decreased to 822 adolescents because of missing data on covariates for the regression model. We used the Statistical Analysis Software (SAS) Version 9.4 (SAS Institute Inc., Cary, NC, USA) for all statistical analyses in this study and used SAS survey procedures to account for the sampling weights.

## 3. Results

Among the 831 adolescents included in the analytic samples, 51.5% were aged 12–14 years (51.5%), 51.1% were males, 51.5% were NH White, 81.2% reported their parents were married or in domestic partnership, 42.0% lived in a household with annual household income of ≥$100,000, 38.5% resided in the South, and 35.3% of parents consumed SSB ≥2 times/day (Table 1). Most adolescents identified that SSB intake is related to cavities (75.4%), weight gain (74.6%), and diabetes (69.7%); however, fewer adolescents identified high blood pressure (31.7%), high cholesterol (25.8%), heart disease (24.6%), and some cancers (18.0%) as related to drinking SSBs (Table 1).

Knowledge of the seven SSB-related health risks significantly varied by certain sociodemographic characteristics (χ^2^ tests, *p* ≤ 0.05). Specifically, knowing that cavities are associated with SSB intake differed significantly by: adolescent age, sex, and race or ethnicity; parent race or ethnicity; as well as annual household income, and census regions of residence. Knowing that weight gain is associated with SSB intake differed significantly by: adolescent race or ethnicity; parent race or ethnicity, and marital status; and annual household income. Knowing that diabetes is associated with SSB intake differed significantly by: adolescent race or ethnicity; parent age, and race or ethnicity; and census regions of residence. Knowing that high blood pressure is associated with SSB intake differed significantly by: adolescent race or ethnicity; parent sex, race or ethnicity, and SSB intake; and annual household income. Knowing that high cholesterol is associated with SSB intake differed significantly by adolescent race or ethnicity and parent race or ethnicity. Knowing that heart disease is associated with SSB intake differed significantly by: adolescent sex; parent age, sex, and marital status; and annual household income. Knowing that some cancers are associated with SSB intake differed significantly by parent sex (Table 1).

Overall, 24.4% of adolescents reported not drinking SSB during the past 7 days, whereas 28.8% of adolescents reported drinking SSB ≥1 time/day (Table 2). Based on bivariate analyses, SSB intake significantly differed by the knowledge that cavities, weight gain, and heart disease are associated with SSB intake (χ^2^ tests, *p* ≤ 0.05). Results of multinomial logistic regression analyses showed that compared to non-SSB consumers, the odds of drinking SSB ≥1 time/day were significantly higher among adolescents who did not know that weight gain (AOR = 2.0), heart disease (AOR = 1.9), or some cancers (AOR = 2.3) are associated with SSB intake vs. adolescents who knew, after adjusting for covariates. Furthermore, compared to non-SSB consumers, the odds of drinking SSB 1–6 times/week were significantly higher among adolescents who did not know that some cancers (AOR = 1.9) are associated with SSB intake vs. adolescents who knew (Table 2).

## 4. Discussion

The present study found that, in 2021, although most US adolescents knew that cavities (75%), weight gain (75/%), and diabetes (70%) are related to drinking SSB, fewer adolescents knew that high blood pressure (32%), high cholesterol (26%), heart disease (25%), and some cancers (18%) are related to drinking SSB. Additionally, after adjusting for covariates, this study found that US adolescents who did not know that weight gain, heart disease, and some cancers are related to drinking SSB had about twice higher odds of consuming SSB at least once per day compared to those who knew.

While the prevalence of having knowledge of health conditions related to drinking SSB was similar among adolescents in different studies, findings were inconsistent on associations between SSB-related knowledge and youth SSB intake [28,31]. For example, an Australian study conducted among 9102 adolescents in 2018 reported results similar to ours. It found that while most Australian adolescents knew that cavities (76%), weight gain/obesity (72%), and diabetes (73%) are related to drinking soft drinks, fewer adolescents knew that heart disease (56%) and cancer (19%) are related to drinking soft drinks [31]. Furthermore, Australian adolescents who knew that cavities, weight gain/obesity, diabetes, and heart disease are related to drinking soft drinks had significantly lower odds of consuming soft drinks at least four cups/week compared to those who did not know. Another study conducted among 982 US adolescents in 2014 (using the same online survey) reported that most adolescents knew cavities (78%), weight gain (75%), and diabetes (61%) are related to drinking SSB [28]. However, inconsistent with our findings, the 2014 study found no association between SSB-related knowledge (about cavities, weight gain, and diabetes) and daily SSB intake among US adolescents after controlling for covariates [28].

Although a direct comparison cannot be made due to differences in study populations, a previous study conducted among US adults in 2014 showed that while most adults knew that cavities (72%), weight gain (80%), and diabetes (74%) are related to drinking SSB, fewer knew that high blood pressure (33%), high cholesterol (24%), and heart disease (32%) are related to drinking SSB [33]. After controlling for covariates, US adults who did not know that heart disease is related to drinking SSB had 1.3 times greater odds for high SSB intake (≥2 times/day) vs. those who knew [33]. However, another study conducted in 2015 among 1000 US Hispanic adults reported no significant association between SSB-related knowledge (i.e., cavities, weight gain, diabetes, high cholesterol, heart disease and high blood pressure) and SSB intake after controlling for covariates [34].

Various studies’ inconsistent findings on associations between SSB-related knowledge and SSB intake might suggest that varying levels of knowledge have different impacts on SSB intake in diverse populations. Potential reasons for the discrepancies are unknown, and the impact of changing knowledge could be tested. Educating adolescents might help reduce youths’ SSB intake, although education alone might not be sufficient. Intervention efforts could evaluate whether increasing certain types of knowledge might influence adolescent SSB intake. Moreover, some studies on adults have found that warning labels that link health effects with consumption have changed adult behaviors. An experimental study showed that pictorial warning levels on SSB (e.g., excess consumption of SSB contributes to type 2 diabetes and heart damage) reduced purchases of SSB among 325 US parents of children aged 2–12 years compared to the control group [35]. Another experimental study among 1360 US adults demonstrated that SSB health warning labels with the marker “WARNING” and octagon shape had the most reactions (such as perceived message effectiveness, fear, and thinking about harms) compared to a rectangle shape with no warning marker or health effect information [36]. Schools can also provide adolescents with health education on SSB reduction. Health education that uses peer mentoring [37,38] and integrates into the core subject curriculum [39] (for instance, science class) has been effective in changing knowledge, attitudes, perceptions, and behaviors among adolescents. In our study, about 3 in 10 US adolescents reported consuming SSB at least once a day in 2021, which is similar to the 2014 study [28]. As daily consumption of SSB among US adolescents remains high, efforts to reduce SSB intake among adolescents may focus on increasing certain types of knowledge that might influence youth SSB intake, and may address other factors associated with SSB intake such as sociodemographic characteristics [21,22,23,24,25], attitudes [26], parent SSB intake [26,28], and availability of SSB at home [29,30].

This study has several limitations. First, the YouthStyles survey is cross-sectional, thus we cannot make inferences about causation. Second, the YouthStyles survey data are self-reported, thus data might be subject to recall bias and social desirability response bias. However, food frequency questionnaires and 24-h recall had moderate agreement based on a previous study [40]. Third, study results might not be generalizable to all US adolescents because participants were randomly chosen from an online panel. However, the data were weighted to be comparable to the distribution from the US Census’ American Community Survey. Lastly, SSB intake was measured in frequency rather than volume, thus the amount of SSB intake cannot be assessed.

## 5. Conclusions

In conclusion, knowledge of SSB-related health risks among US adolescents varied by condition, ranging from 18% for some cancers to 75% for cavities and weight gain. Most adolescents identified cavities, weight gain, and diabetes as related to drinking SSB; fewer adolescents identified high blood pressure, high cholesterol, heart disease, and some cancers as being related to drinking SSB. Not knowing that weight gain, heart disease, and some cancers are associated with SSB intake increased the odds of drinking SSB daily. Intervention efforts could focus on increasing certain types of knowledge that might influence SSB intake in youth—to support their health.

## Figures and Tables

**Table 1:** Characteristics of respondents and their associations with adolescent knowledge of health conditions related to sugar-sweetened beverage (SSB) intake ^a^ among US adolescents, YouthStyles survey, 2021.

	Weighted % ^b^ ± Standard Error							
		Adolescent Knowledge of Health Conditions Related to SSB Intake (Answering Yes) ^c^						
Characteristic	All	Cavities	Weight Gain	Diabetes	High Blood Pressure	High Cholesterol	Heart Disease	Some Cancers
Total sample (unweighted, *n* = 831)	100	75.4 ± 1.9	74.6 ± 1.9	69.7 ± 2.0	31.7 ± 2.0	25.8 ± 1.9	24.6 ± 1.8	18.0 ± 1.6
Adolescent age								
12–14 years	51.5 ± 2.1	**79.1 ± 2.4**	76.2 ± 2.6	67.8 ± 2.8	30.7 ± 2.8	24.8 ± 2.6	24.8 ± 2.6	18.4 ± 2.3
15–17 years	48.5 ± 2.1	**71.5 ± 2.9**	72.9 ± 2.8	71.7 ± 2.7	32.8 ± 2.8	26.8 ± 2.7	24.4 ± 2.5	17.7 ± 2.3
Adolescent sex								
Male	51.1 ± 2.1	**71.7 ± 2.8**	73.7 ± 2.7	66.7 ± 2.8	28.6 ± 2.7	22.2 ± 2.6	**21.0 ± 2.4**	17.6 ± 2.3
Female	48.9 ± 2.1	**79.3 ± 2.5**	75.6 ± 2.7	72.8 ± 2.7	35.0 ± 2.9	29.6 ± 2.7	**28.3 ± 2.6**	18.5 ± 2.3
Adolescent race or ethnicity (*n* = 830)								
NH Black	12.8 ± 1.7	**59.7 ± 7.6**	**56.0 ± 7.6**	**52.0 ± 7.5**	**14.6 ± 4.7**	**16.2 ± 4.9**	19.9 ± 5.5	16.6 ± 5.7
Hispanic	25.0 ± 2.0	**76.6 ± 4.3**	**79.7 ± 3.9**	**80.4 ± 3.6**	**43.2 ± 4.9**	**36.5 ± 4.8**	30.4 ± 4.5	20.4 ± 3.9
NH Other/Multiracial	10.7 ± 1.3	**76.2 ± 5.1**	**79.4 ± 5.6**	**74.6 ± 5.1**	**41.2 ± 6.3**	**28.5 ± 5.5**	24.0 ± 5.3	18.8 ± 4.9
NH White	51.5 ± 2.2	**78.5 ± 2.0**	**75.7 ± 2.1**	**67.7 ± 2.3**	**28.3 ± 2.2**	**22.2 ± 2.1**	22.8 ± 2.0	16.8 ± 1.8
Parent age								
18–34 years	8.3 ± 1.5	76.2 ± 8.9	58.5 ± 9.9	**55.9 ± 9.7**	21.6 ± 8.2	25.8 ± 8.7	**8.5 ± 4.2**	15.3 ± 6.7
35–44 years	44.9 ± 2.1	75.8 ± 2.9	75.9 ± 2.8	**74.7 ± 2.7**	34.1 ± 3.0	27.6 ± 2.9	**26.6 ± 2.8**	18.4 ± 2.4
≥45 years	46.8 ± 2.1	74.9 ± 2.5	76.2 ± 2.5	**67.3 ± 2.8**	31.3 ± 2.6	24.0 ± 2.4	**25.5 ± 2.5**	18.1 ± 2.3
Parent sex								
Male	37.9 ± 2.0	71.5 ± 2.9	74.9 ± 2.8	68.3 ± 2.9	**37.2 ± 3.1**	27.8 ± 2.9	**29.4 ± 2.9**	**24.0 ± 2.8**
Female	62.1 ± 2.0	77.8 ± 2.5	74.4 ± 2.6	70.5 ± 2.6	**28.4 ± 2.5**	24.6 ± 2.4	**21.7 ± 2.2**	**14.4 ± 1.9**
Parent race or ethnicity								
NH Black	13.6 ± 1.8	**58.3 ± 7.4**	**50.9 ± 7.4**	**53.1 ± 7.3**	**14.5 ± 4.3**	**14.9 ± 4.2**	16.1 ± 4.6	15.3 ± 5.3
Hispanic	18.1 ± 1.9	**72.9 ± 5.4**	**74.3 ± 5.1**	**78.1 ± 4.5**	**43.1 ± 5.9**	**38.0 ± 5.8**	31.6 ± 5.4	20.7 ± 4.5
NH Other/Multiracial	7.9 ± 1.1	**75.6 ± 6.1**	**82.2 ± 5.5**	**75.8 ± 5.8**	**41.6 ± 7.5**	**26.6 ± 6.5**	21.5 ± 6.2	15.2 ± 5.6
NH White	60.4 ± 2.2	**80.0 ± 1.8**	**79.0 ± 1.8**	**70.0 ± 2.2**	**30.9 ± 2.2**	**24.5 ± 2.1**	24.8 ± 2.0	18.2 ± 1.8
Parent education								
≤ High school/GED	29.3 ± 2.1	70.2 ± 4.4	69.3 ± 4.5	64.8 ± 4.3	31.1 ± 4.1	28.1 ± 4.1	19.1 ± 3.2	17.3 ± 3.4
Some college	29.7 ± 1.9	75.4 ± 3.3	76.6 ± 3.3	73.4 ± 3.3	28.4 ± 3.4	24.1 ± 3.2	24.6 ± 3.3	17.9 ± 2.8
≥ College graduate	41.0 ± 2.0	79.1 ± 2.3	76.9 ± 2.4	70.4 ± 2.7	34.6 ± 2.9	25.4 ± 2.6	28.5 ± 2.7	18.6 ± 2.3
Parent marital status								
Married/domestic partnership	81.2 ± 1.8	76.4 ± 2.0	**77.8 ± 1.9**	71.2 ± 2.0	32.4 ± 2.1	25.5 ± 2.0	**26.5 ± 2.0**	18.5 ± 1.8
Not married	18.8 ± 1.8	71.3 ± 5.4	**60.7 ± 5.6**	63.2 ± 5.5	29.0 ± 5.0	26.8 ± 4.8	**16.6 ± 3.4**	16.1 ± 4.0
Annual household income								
<$35,000	19.1 ± 2.0	**62.4 ± 6.1**	**60.5 ± 6.1**	64.6 ± 5.9	**17.4 ± 4.4**	20.9 ± 5.0	**10.8 ± 3.2**	14.0 ± 4.1
$35,000–$74,999	24.8 ± 1.8	**76.6 ± 3.6**	**80.6 ± 3.6**	69.4 ± 3.8	**36.6 ± 4.2**	28.3 ± 3.9	**26.6 ± 3.8**	21.9 ± 3.5
$75,000–$99,999	14.1 ± 1.3	**76.7 ± 4.1**	**76.7 ± 4.1**	76.8 ± 4.0	**35.8 ± 4.9**	31.0 ± 4.8	**31.1 ± 4.9**	15.6 ± 3.5
≥$100,000	42.0 ± 2.0	**80.2 ± 2.3**	**76.7 ± 2.4**	69.7 ± 2.7	**34.0 ± 2.9**	24.8 ± 2.6	**27.5 ± 2.7**	18.4 ± 2.3
Census regions of residence								
Northeast	16.3 ± 1.5	**65.7 ± 4.9**	74.0 ± 4.5	**62.3 ± 4.9**	31.7 ± 4.8	28.2 ± 4.7	26.9 ± 4.4	17.7 ± 4.3
Midwest	21.4 ± 1.6	**75.7 ± 3.5**	72.3 ± 3.8	**62.7 ± 4.1**	28.1 ± 3.6	24.9 ± 3.5	21.7 ± 3.2	19.1 ± 3.0
South	38.5 ± 2.1	**74.9 ± 3.4**	72.5 ± 3.5	**73.2 ± 3.3**	31.2 ± 3.4	23.6 ± 3.1	23.4 ± 2.9	16.3 ± 2.6
West	23.8 ± 1.8	**82.6 ± 3.3**	80.4 ± 3.4	**75.2 ± 3.5**	35.9 ± 4.2	28.4 ± 4.1	27.5 ± 4.0	20.2 ± 3.5
Parent SSB intake (*n* = 823)								
0 times/day	12.7 ± 1.4	78.7 ± 5.1	75.2 ± 5.5	66.7 ± 5.9	**28.4 ± 5.0**	20.0 ± 4.5	21.6 ± 4.8	13.3 ± 3.7
>0 to <1 time/day	25.1 ± 1.8	79.9 ± 3.3	77.1 ± 3.3	75.1 ± 3.2	**40.2 ± 4.1**	26.6 ± 3.6	25.9 ± 3.4	16.6 ± 2.8
1 to <2 times/day	26.8 ± 1.9	75.8 ± 3.7	74.0 ± 3.8	68.2 ± 3.8	**25.2 ± 3.3**	25.3 ± 3.5	20.7 ± 3.2	16.2 ± 2.8
≥2 times/day	35.3 ± 2.1	71.6 ± 3.5	73.9 ± 3.5	69.5 ± 3.5	**32.6 ± 3.5**	27.3 ± 3.4	28.1 ± 3.3	22.3 ± 3.3

SSB: sugar-sweetened beverage; GED: General Educational Development; NH: non-Hispanic. ^a^ Determined by the question, “Which of the following conditions do you think are related to drinking sugary drinks, such as regular sodas, fruit drinks (e.g., Kool-Aid, lemonade), sports or energy drinks (e.g., Gatorade, Red Bull), and sweetened teas?” ^b^ Weighted percent may not add up to 100% because of rounding. ^c^ χ^2^ tests were used for each variable to examine differences across categories. *p* ≤ 0.05 was bolded.

**Table 2:** Bivariate and multivariate associations between sugar-sweetened beverage (SSB) intake ^a^ during the past 7 days and knowledge of health conditions related to SSB intake among US adolescents ^b^, YouthStyles survey, 2021.

		Bivariate Analysis of Adolescent SSB Intake (Unweighted *N* = 831)				Multinomial Logistic Regression Analysis of Adolescent SSB Intake	
		Weighted % ^c^ ± Standard Error				Adjusted OR (95% CI) ^d^	
Knowledge of Conditions Related to SSB Intake	All	None	1–6 Times/Week	≥1 Time/Day	*p* Value ^e^	1–6 Times/Week	≥1 Time/Day
Total sample	100	24.4 ± 1.8	46.8 ± 2.1	28.8 ± 2.0			
Cavities					0.05		
No	24.6 ± 1.9	16.8 ± 3.5	48.4 ± 4.5	34.8 ± 4.5		1.73 (0.97, 3.09)	1.67 (0.89, 3.14)
Yes	75.4 ± 1.9	26.9 ± 2.1	46.3 ± 2.4	26.8 ± 2.2		Reference	Reference
Weight gain					0.01		
No	25.4 ± 1.9	18.6 ± 3.4	42.4 ± 4.4	39.0 ± 4.6		1.35 (0.79, 2.29)	2.00 (1.11, 3.61) ^f^
Yes	74.6 ± 1.9	26.4 ± 2.1	48.3 ± 2.4	25.3 ± 2.1		Reference	Reference
Diabetes					0.26		
No	30.3 ± 2.0	20.4 ± 3.2	46.9 ± 3.8	32.6 ± 3.7		1.29 (0.78, 2.16)	1.49 (0.86, 2.58)
Yes	69.7 ± 2.0	26.2 ± 2.2	46.7 ± 2.5	27.1 ± 2.4		Reference	Reference
High blood pressure					0.70		
No	68.3 ± 2.0	23.4 ± 2.2	47.3 ± 2.6	29.3 ± 2.4		1.27 (0.80, 2.02)	1.19 (0.69, 2.07)
Yes	31.7 ± 2.0	26.6 ± 3.3	45.7 ± 3.7	27.6 ± 3.5		Reference	Reference
High cholesterol					0.26		
No	74.2 ± 1.9	22.8 ± 2.1	47.0 ± 2.4	30.2 ± 2.3		1.39 (0.86, 2.26)	1.63 (0.90, 2.94)
Yes	25.8 ± 1.9	29.1 ± 3.9	46.2 ± 4.2	24.7 ± 3.8		Reference	Reference
Heart disease					0.03		
No	75.4 ± 1.8	22.4 ± 2.1	46.3 ± 2.4	31.4 ± 2.4		1.46 (0.91, 2.34)	1.90 (1.06, 3.39) ^f^
Yes	24.6 ± 1.8	30.6 ± 3.8	48.4 ± 4.1	21.0 ± 3.3		Reference	Reference
Some cancers					0.10		
No	82.0 ± 1.6	22.7 ± 2.0	47.3 ± 2.3	30.1 ± 2.2		1.85 (1.10, 3.11) ^f^	2.27 (1.19, 4.32) ^f^
Yes	18.0 ± 1.6	32.3 ± 4.5	44.7 ± 4.9	23.0 ± 4.5		Reference	Reference

SSB: sugar-sweetened beverage; CIs: confidence intervals; ORs: odds ratios. ^a^ Adolescent SSB intake was measured using one question, and SSB included sodas, fruit drinks, sports or energy drinks, and other SSB (excluding 100% fruit juice or diet drinks). ^b^ Determined by the question, “Which of the following conditions do you think are related to drinking sugary drinks, such as regular sodas, fruit drinks (e.g., Kool-Aid, lemonade), sports or energy drinks (e.g., Gatorade, Red Bull), and sweetened teas?” ^c^ Weighted percent may not add up to 100% because of rounding. ^d^ The outcome variable was SSB, and the exposure variables were knowledge of health conditions related to SSB intake. The reference category for SSB intake was none. Because of potential collinearity issues among exposure variables, seven separate multinomial logistic regression models were fit to include each exposure variable and controlled for adolescent age, sex, and race or ethnicity; parent age, sex, race or ethnicity, education, and marital status; annual household income, census regions of residence, and parent SSB intake. Sample size for regression models was *n* = 822 for adolescent knowledge of health conditions related to SSB intake. ^e^ χ^2^ tests were used for each variable to examine differences across categories. ^f^ Considered statistically significant based on 95% CI.

## Data Availability

Data sharing is not applicable to this article.
